# Single step PCR for the identification of Low Density Lipoprotein Receptor (LDL-R) gene mutations

**DOI:** 10.12669/pjms.304.4711

**Published:** 2014

**Authors:** Samia Perwaiz Khan, Rubina Ghani, Zia Yaqub

**Affiliations:** 1 Samia Perwaiz Khan, Professor of Pharmacology, Department of Pharmacology, Ziauddin University, Karachi, Pakistan.; 2 Rubina Ghani, Associate Professor, Baqai Medical University,; 3 Zia Yaqub, National Institute of Cardiovascular Diseases, Karachi, Pakistan.

**Keywords:** Familial hypercholesterolemia (FH), low density lipoprotein receptor (LDL-R), Direct PCR (polymerase chain reaction), Kappa Blood Kit

## Abstract

***Background and Objective:*** This study was conducted to determine the common mutation of low density lipoprotein receptor in patients with familial hypercholesterolemia (FH) in our population and identify the different point mutation in the LDL-receptor gene. The main aim of this study was to reduce the cost of PCR without extracting DNA and do the diagnosis at single step.

***Methods:*** This study was carried out in the period of one year, from 2009- 2011. All the patients selected for this study were from Dr. Ziauddin Hospital, National Institute of Cardiovascular Diseases, and Dr. Rubina Ghani’s Pathological & Molecular Laboratories. While collecting the blood sample, the patients were in overnight fasting condition. The clinical and biochemical analysis was performed on hyperlipidemic patients (n=120) to determine the frequency of familial hypercholesterolemia in our population. After lipid profile the patients were selected and direct multiplex PCR (Polymerase chain reaction) was performed from whole blood collected in a single tube using forward and reverse primers of exons 3, 4, 9 and 14 of without extracting DNA.

***Results:*** Genomic DNA was extracted from blood samples as well as direct whole ETDA blood of healthy control group and hypercholesterolemia patients to detect mutations in exons 3, 4, 9, and 14 of the LDLR gene, with modification in the technique by using type-specific primers. These results for exon 4 mutation were confirmed by DNA sequencing.

***Conclusion: ***Screening method based on PCR by using Kappa direct PCR could be a faster and cheaper method with least contamination for screening a large number of FH patients for mutation of LDLR gene.

## INTRODUCTION

Familial hypercholesterolemia (FH) clinical characteristics include elevated LDL cholesterol levels, tendon xanthomas and increased risk of premature coronary artery diseases.^1^^,^^2^ At molecular level it is autosomal dominant diseases due to mutation of LDL receptor gene, more than 900 different LDL-R gene mutations have been reported world wide.^3^ Structural rearrangements are responsible for 5% mutation in heterozygous familial hypercholesterolemia.^4^

The mutations of LDL-R gene identified are reported to result in a typical clinical picture of Familial hypercholesterolemia, with very high serum LDL-C, tendon xanthomas and premature coronary heart disease.^5^

Common mutations of LDL receptor have influence on serum cholesterol levels in heterozygous FH. Patients with homozygous FH have shown inter individual variation in serum total cholesterol and early onset of coronary heart disease.^6^

Southern blotting followed by hybridization with LDL-R probes have been used to screen for major rearrangements.^7^ Another study has identified two LDL-receptor mutations causing familial hypercholesterolemia in Indian subjects by a simplified rapid PCR- heteroduplex method.^8^

The diagnosis of FH is made on raised LDL-C levels in individuals and family members, although clinical features and family history does not give exact diagnosis in every individual. The DNA based genetic test for FH is best for the identification of affected persons in a family. The genetic screening of a family to find new patients with FH is faster and more reliable compared with a biochemical screening.^9^ At present number of LDL-R gene mutations are reported (24%) small DNA rearrangements and (11%) large DNA rearrangements.^10^ The large DNA rearrangement is associated with Alu element.

KAPA Blood DNA Polymerase is the first DNA polymerase engineered specifically for the amplification of DNA directly from whole blood. The enzyme is available in two optimized, easy-to-use 2x PCR mixes, containing all components required for whole Blood PCR, except primers and template (blood). Using KAPA Blood PCR Kits, DNA fragments may be amplified directly from reactions containing 1 - 20% (v/v) whole human blood without pretreatment of blood samples or DNA isolation, reducing contamination, time and cost of genetic testing.^11^

## METHODS

Total 120 hypercholesterolemia patients were diagnosed by performing lipid profiles after twelve hours fast and with family history of premature coronary heart diseases. Patients were included from Dr. Ziauddin Hospital and National Institute of Cardiovascular Diseases, Karachi, Pakistan. Written consent was taken from all these subjects.

Polymerase chain reaction (PCR) amplification of type specific primers lead to rapid detection of point mutations in exon 4, of the low density lipoprotein receptor gene in hypercholesterolemia patients.

DNA extraction The genomic DNA was extracted from whole blood collected in EDTA tubes and the DNA extraction according to standard technique^12^^,^^13^ was performed, by following the Epicenter DNA Purification Kit (Cat No.MCD85201) procedure. The multiplex PCR was performed in a single tube using mutation specific primers. The PCR reactions contained 10mM Tris/HCl, 50mM KCl, 1.5mM MgCl2, 50 pM of each nucleotide. A total 15 μl final PCR reaction volume was used for this purpose. The reaction volume was 0.5 micrograms of the DNA template, 10 pmol of each of the eight primers, 2. 5 unit Taq DNA polymerase, and 0.2 mM of each dNTP in a solution of 10 mM Tris- HC1, 50mM KCl and 1.2mM MgCl2 (Promega, USA). Direct-PCR Direct PCR was done for twenty samples only to compare two commercially available DNA-polymerases from KAPA Biosystems and Promega Gotaq master mix technique for mutation screening in the LDL receptor gene disorder such as familial hypercholesterolemia (FH). Approaches for mutation screening are required for the development of cost-effective genetic tests. The EDTA sample was used for LDL genotyping for the detection of mutation in different exons and PCR was performed. The lipid profile parameters were determined including total cholesterol, LDL-C, HDL-C and triglycerides. Genotyping was performed for rapid, reproducible and direct molecular diagnostic assays utilizing direct polymerase chain reaction (PCR) in two phases. Phase one PCR for LDL-R gene was done with DNA extraction by using DNA extraction kits (e.g. Epicenter, USA).

Second phase, the direct-PCR technique (i.e. without DNA extraction step from whole blood) using commercial kits (i.e. KAPA Blood PCR Direct PCR Buffer) using specific set of, multiplex primers and PCR was performed in a single tube. In Kapa direct PCR buffer, 5µl of whole blood collected in EDTA, was added, with 400nM of dNTP mix, 10nM of DMSO and 30 pg mole/l of sets primer were also added to the PCR buffer. The thermal cycling condition consisted of initial denaturation at 94oC for 5 minute continuing to 35 cycles of denaturation 94^o^C for 45 seconds, primer annealing at 55 ^o^C for 45 seconds and extension at 72 ^o^C for 1.5 minute with the final extension at 72 ^o^C for 7 minutes. Electrophoresis Fifteen micro liters of the PCR products were removed and mixed with 3 μL of a loading buffer and then loaded on 2% agarose gel. The gel was set at 100 volts for one hour and then stained with ethidium bromide. After staining, the bands could be seen under UV light. The different mutations were characterized with 100bp DNA ladder for common four mutations of LDL receptor gene. DNA sequencing (exon 4) Mutation band of exon 4 at 431bp were separated out for sequencing of cases of familial hypercholesterolemia. Automated DNA sequencing with coloured probes green for adeninie, black for guanine, red for thymine and blue for cytosine was done to screen for mutation of exon 4. This sequencing was performed at Centre of molecular and biological excellence (CEMB), Lahore.

## RESULTS

One Hundred and twenty hyperlipidemics from Dr. Ziauddin Hospital and National Institute of Cardiovascular Diseases with raised LDL-C above 160 mg/d were included in the study. However this was a concentrated population of hundred and twenty hyperlipidemic patients, as all of these were selected from the cardiovascular unit of the hospital OPD. In these cases, forty two cases were found to have high LDL-C, xanthalasmias, tendon xanthomas and arcus cornea with exon 3, 4 mutation of LDLR gene. Other patients had high LDL- cholesterol and family history of coronary artery diseases. In addition to cholesterol measurement with hyperlipidemia, clinical signs with tendon xanthomas and family history are the criteria for Classical cases of hypercholesterolemia patients along LDLR gene mutation. Mutation LDLR gene (exon 4) was determined by Direct PCR Fig.1a, PCR after DNA extraction is shown in Fig.1b. The amplified product for exon 4 was confirmed after the DNA sequences as shown in Fig. 2a and 2b. Comparison of cost of Direct PCR and PCR with DNA extraction is given in Table-I.

## DISCUSSION

Satisfactory polymerase chain reaction (PCR) was done using direct PCR kit and with DNA extractions. Both methods of PCR have shown similar results in this study. PCR amplifications of the hemochromatosis gene and apo E genes were obtained using freshly drawn untreated blood samples tested by PCR.^14^ Genetic diagnostic tests will assist in the identification of FH while improving cardiovascular risk prediction, prevention of disease and treatment efficacy.^15^^,^^16^ About 35% classical and 65% probable cases of heterozygous FH were observed with mutation at exon 3 and 4 in Pakistani population.^17^

**Fig.1a:**
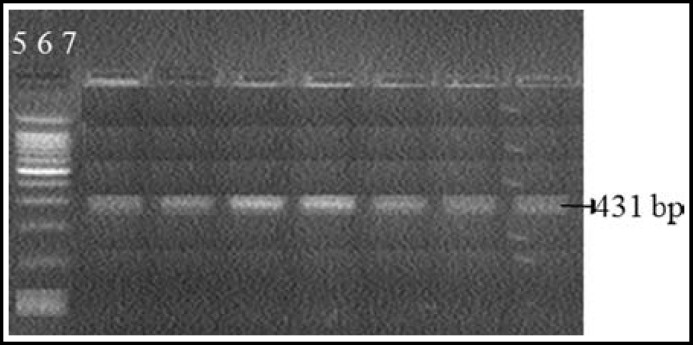
PCR without DNA extraction (Show mutation in exon 4)

**Fig.1b:**
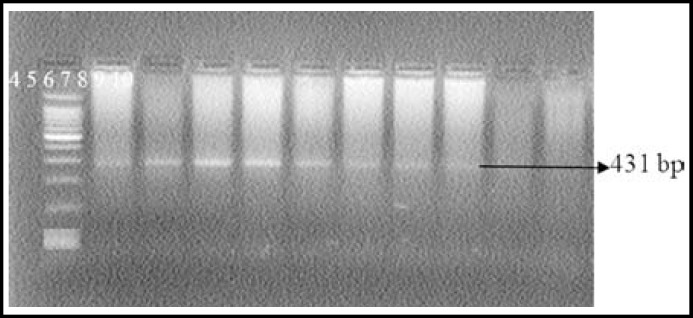
PCR with DNA extraction (Show mutation in exon 4)

**Fig.2a:**
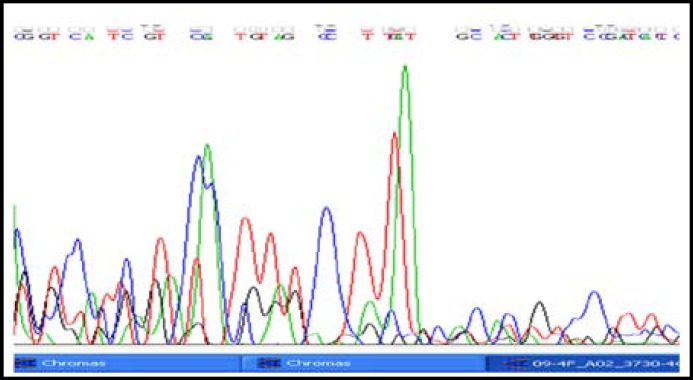
Exon 4 – Forward

**Fig.2b:**
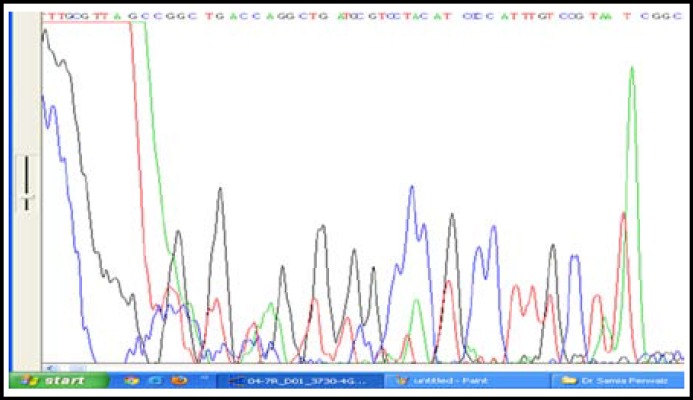
Exon 4 –Reverse

**Table-I:** This table shows the cost difference between two ways of PCR performed for identification of low density lipoprotein receptor (LDL-R) gene mutations

***Items***	***Conversional PCR Cost***	***Direct PCR*** ***Kappa KIt***
DNA Extraction	Rs 500	Not used
Master Mix +dNTPS + Primers	Rs 400	Rs 250
DEMSO	Not used	Rs 50
Agarose gel	Rs 200	Rs 200
Total	Rs 1100	Rs 500

Direct amplification of genomic DNA from whole blood without DNA isolation was done by using the PCR buffer with a higher pH, which was optimized as pH 9.1–9.6. Direct PCR on blood treated with various anticoagulants showed that the buffer worked well with the blood treated by citrate, EDTA, or heparinate. DNA fragments with different lengths could be efficiently amplified directly from various forms of blood.^18^ Direct amplification from fresh or frozen whole blood, does not require DNA extraction in genetic testing. Rubina et al. also used direct PCR for rapid and reproducible results for diagnosis of hepatitis B, Typhi S and Beta Thalassemia by Kapa blood PCR mix.^19^

Direct blood PCR is easy, rapid, economical, reproducible and specific method for the detection of meningococci in blood samples, it also could facilitate the large-scale screening of various medical conditions.^20^ With the use of direct PCR contamination, time and cost of genetic testing are reduced. The results were similar with routinely used detection methods.

## CONCLUSIONS

The point mutation on exon 4 of LDLR gene was most common. It was also concluded that screening method based on PCR by using Kappa direct PCR could be a faster and cheaper method with least contamination for screening a large number of FH patients for mutation of LDLR gene.

## Authors Contributions:

**SPK: **Principle investigator, concept and design, data collection, PCR and sequencing, statistical analysis and interpretation of data.

**RG: **Data collection, PCR and sequencing, statistical analysis and interpretation of data.

**YZ:** Familial hypercholesterolemia patients who participated in this study were diagnosed by him from OPD and ward.
